# A Review of the Public Health Challenges of *Salmonella* and Turtles

**DOI:** 10.3390/vetsci7020056

**Published:** 2020-04-27

**Authors:** Hamid Reza Sodagari, Ihab Habib, Majedeh Pakzad Shahabi, Narelle A. Dybing, Penghao Wang, Mieghan Bruce

**Affiliations:** 1 School of Veterinary Medicine, College of Science, Health, Engineering and Education, Murdoch University, Perth 6150, Australia; hr.sodagari@gmail.com (H.R.S.); n.dybing@murdoch.edu.au (N.A.D.); p.wang@murdoch.edu.au (P.W.); mieghan.bruce@murdoch.edu.au (M.B.); 2 Veterinary Medicine Department, College of Food and Agriculture, United Arab Emirates University (UAEU), Al Ain P.O. Box 1555, UAE; 3 Faculty of Veterinary Medicine, Ferdowsi University of Mashhad, Mashhad 1696, Iran; majedehpakzad@gmail.com

**Keywords:** *Salmonella*, turtles, human salmonellosis, One Health, zoonosis

## Abstract

Non-typhoidal *Salmonella* serovars are recognized as zoonotic pathogens. Although human salmonellosis is frequently associated with ingestion of contaminated foods of animal origin, contact with animals may also be a significant source of *Salmonella* infection, especially contact with turtles, which have shown to be an important reservoir of *Salmonella,* specifically through their intestinal tracts. Turtles are among the most common reptiles kept as house pets that may pose a public health risk associated with *Salmonella* exposure, especially among infants and young children. This review discusses the literature reporting the link between turtles and *Salmonella* as well as turtle-associated human salmonellosis in the last ten years. In most outbreaks, a high proportion of patients are children under five years of age, which indicates that children are at the greatest risk of turtle-associated salmonellosis. Therefore, turtles should not be preferred as recommended pets for children under five years of age. Reducing turtle stress to minimise *Salmonella* shedding as well as providing client education handouts at the points of sale of these animals may reduce the risk of transmitting such significant pathogen to humans. Further studies are required to investigate the role of both direct contact with turtles as well as indirect contact through cross-contamination in the transmission of turtles-associated *Salmonella* to humans.

## 1. Introduction

Non-typhoidal *Salmonella* serovars are recognized as zoonotic pathogens affecting both animals and humans. There are over 2500 *Salmonella* serovars, of which 2000 of them can affect humans [1]. This pathogen can cause mild to severe disease with clinical symptoms, including fever, diarrhoea, and vomiting. Although salmonellosis is usually a self-limiting illness, it can be life-threatening in high-risk patients, such as babies, the elderly and immunocompromised people [2]. It has been reported that *Salmonella* are responsible for 1.2 million cases of illness and 450 deaths in humans annually in the United States [3]. Although human salmonellosis is frequently associated with ingestion of contaminated foods of animal origin, contact with animals may also be a significant source of *Salmonella* infection [4]. Hale et al. (2012) estimated that 127,155 of 445,213 human illnesses caused by seven groups of zoonotic pathogens annually have been attributed to non-typhoidal *Salmonella* serovars related to animal exposure [5].

The intestinal tract of reptiles is a major reservoir of *Salmonella* [6]. Reptiles can carry *Salmonella* without showing any clinical symptoms and shed this pathogen in their faeces [7]. The number of reptiles housed as pets has become increasing worldwide [8,9]. Several human salmonellosis cases have been attributed to reptiles directly or indirectly due to the high number of such animals kept as house pets [1,10]. Numerous *Salmonella* serovars have been attributed to the reptile-associated salmonellosis, comprising *Salmonella enterica* (*S. enterica*) serovars Paratyphi B var Java, Poona, Pomona, Marina, Stanley, Litchfield, and Newport, as well as the most commonly reported *S. enterica* serovars Typhimurium and Enteritidis [6,7,11,12,13,14]. Among different reptiles, turtles are more commonly considered house pets, and they are among the most commonly kept pet reptiles for children [15]. Small turtles are more likely to be given as pets to children because they are safe, attractive, inexpensive, and slow-moving, compared to other pet reptiles, such as snakes and iguanas [7,16]. The common practice of children keeping turtles as house pets may pose a public health risk associated with *Salmonella*, especially for infants and young children [17]. The turtles are small enough to be kissed and held by children, which increases the likelihood of direct transmission of *Salmonella*. In addition, indirect transmission of this pathogen can occur through cross-contamination by cleaning turtle habitats in a kitchen sink or bathtub [18]. The first report of turtle-associated human salmonellosis goes back to 1963 [19]. There are also several reports related to the transmission of *Salmonella* to humans through direct or indirect contact with turtles in the last decade [5,9,20]. Therefore, here we present an updated review that was designed to investigate the research and evidence related to *Salmonella* and turtles as well as their link to human salmonellosis in the last ten years in different parts of the world. Furthermore, different *Salmonella* serovars, turtle species, and human cases are examined.

## 2. Materials and Methods

**Figure 1:**
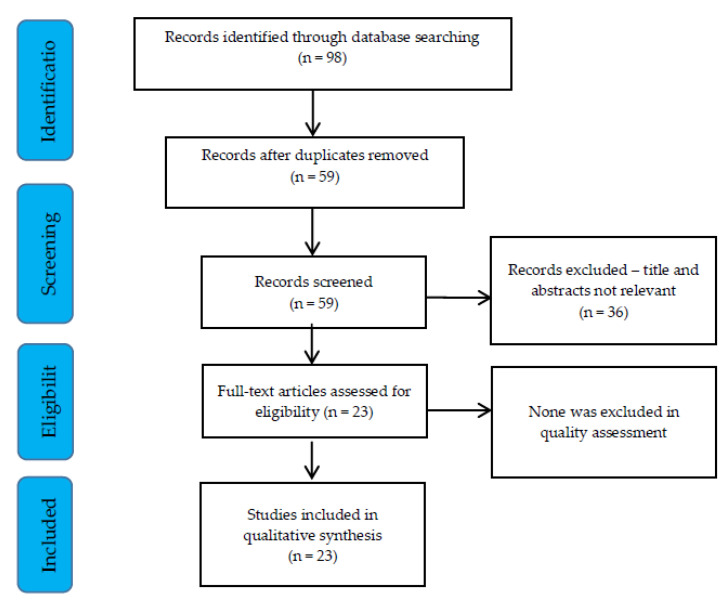
Overview of the search strategy and process of articles’ inclusion.

The search strategy used in the present review was based on the following criteria: (a) the databases PubMed and Web of Science were searched for articles written in English or with English abstracts over the past decade, using the keywords of: (*Salmonella* OR salmonellosis) AND (turtle OR turtles OR reptiles); (b) screening the articles by reading their titles and abstracts in order to exclude non-relevant research if they did not refer to human salmonellosis or if they were review or letter articles as and were not related to the isolation of *Salmonella* in turtles or turtle-associated human salmonellosis; (c) articles were included if they were population studies investigating human cases of salmonellosis associated with turtles (e.g., clinical or outbreak cases) or to detection of *Salmonella* in turtles. Figure 1 indicates the systematic approach to articles’ inclusion and exclusion.

## 3. Most Popular Pet Turtles

The pet trade is growing globally, and amphibians and reptiles are the most common among pet species around the world [21]. Emydidae, a family of turtles that includes close to 50 species in 10 genera, is among the most recognized reptile taxon in the world [21]. Pond sliders (*Trachemys scripta*) are the main species of this family, which is divided into three subspecies [21]. Among these three subspecies, the most recognized is the red-eared slider (*Trachemys scripta elegans*), which is the most common pet turtle around the world and has been found to be a significant source of turtle-associated human salmonellosis since 1963 [8]. Table 1 shows the most popular pet turtles worldwide [22].

## 4. *Salmonella* Contamination of Turtles and Turtle-Associated Human Salmonellosis around the World

### 4.1. North America

Turtles are recognized as the most popular pet reptiles in the United States [23]. Despite a prohibition on the distribution of small turtles (carapace length < 10.2 cm or 4 inches) in the United States since 1975, they are still available legally for scientific, educational, or exhibition purposes and are sold illegally at fairs, discount stores, flea markets outside of sporting events, or at parks [24,25] Approximately 1.4 million human salmonellosis cases occur annually in the United States, of which 74,000 have been associated with direct or indirect exposure to reptiles and amphibians [11]. Turtle-associated salmonellosis in the U.S. has not only persisted but has increased, according to a surge in the number of salmonellosis outbreaks in the last decade, which has brought increased attention to this long-standing public health issue [23]. Eight multistate outbreaks involving 473 illnesses were reported in the U.S. in 2012, and *S. enterica* serovars Sandiego, Poona, Pomona, Newport, and Typhimurium were the five identified serovars in these outbreaks [23]. The highest number of cases and hospitalisations were attributed to one of these outbreaks caused by *S. enterica* serovar Pomona. However, no deaths were reported from these eight outbreaks. The report on the outbreaks indicated that 8% of those affected had been exposed to small turtles. Those affected were less knowledgeable on the link between reptiles and salmonellosis than individuals affected in previous turtle-associated outbreaks [18]. Several high-risk behaviours, including kissing turtles, cleaning turtle habitats in kitchen sinks, and allowing turtles access to kitchen countertops and other places where food is prepared and consumed, were reported as creating the main transmission routes of *Salmonella* to humans in the 2012 outbreaks [23].

In 2014, pet turtle-associated salmonellosis was reported in 12 states [23]. Most of the cases were observed in children less than one year of age. The most frequently detected infectious agent was *S. enterica* serovar Poona. No deaths were reported, although a few of those who contracted the disease were hospitalised. Interestingly, not all patients mentioned direct contact with turtles, suggesting the potential role of indirect transmission in turtle-associated salmonellosis outbreaks, which should be more closely examined [20].

In 2015, there was a report of an *S. enterica* serovar Sandiego infection in a child who had acquired a small turtle at an Alabama flea market. Four multistate *Salmonella* outbreaks were also reported in that year [24]. The identified serovars in these outbreaks (*S. enterica* serovar Sandiego and *S. enterica* serovar Poona) had been linked to small turtles in previous outbreaks [18,20]. The high proportion of patients in these outbreaks are younger than five years old indicating that children are still the main group affected by turtle-associated salmonellosis. This finding emphasises the need to educate this susceptible population regarding the risk of *Salmonella* transmission from companion small turtles and other reptiles [25].

Another salmonellosis outbreak reported in 19 states in 2017 was caused by *S. enterica* serovar Agbeni. More than half of the cases involved direct or indirect contact with pet turtles, including small turtles [26]. The same *Salmonella* serovar (*S. enterica* serovar Agbeni) was identified previously from a turtle in 2015 and another human outbreak in 2016 (CDC, unpublished data, 2016). Interestingly, a higher frequency of hospitalisations (48%) was attributed to this outbreak compared to multistate foodborne pathogen outbreaks (27%) and recent turtle-associated salmonellosis outbreaks (28–33%) [20,25,27] (Table 2).

The geographic distribution of patients affected by the salmonellosis outbreak was different from the previous outbreaks, indicating the need to better understand turtle breeding and distribution in the United States. Sales of small turtles have been banned in 18 states of this country. Some states incorporate the federal standard by reference while others explicitly ban the sale of small turtles below a certain size [28].

### 4.2. South America and Caribbean Island

Pet turtle-associated *Salmonella* also caused gastroenteritis in three infants in Chile [29]. *S. enterica* subsp. *enterica* serovars Montevideo, Newport, and Pomona were the identified *Salmonella* serovars in these cases. In two of the cases, *Salmonella* was recovered from the patients’ stools and the turtles’ droppings [29] (Table 2).

Considering the significance of turtle-associated salmonellosis in human health several studies have been conducted in different parts of the world in order to improve the knowledge about the rate of *Salmonella* contamination in turtles. In Saint Kitts, three different sea turtle species can be found year round, including marine environment turtle species (hawksbill, *Eretmochelys imbricata,* and green turtles, *Chelonia mydas*), and seasonal nester species (leatherback, *Dermochelys coriacea*) [30]. Little is known about the risk of *Salmonella* transmission from wild sea turtles to humans, and it has only been investigated in a few studies [31,32]. One of these studies was conducted on the island of Saint Kitts on nesting leatherback sea turtles. *Salmonella enterica* was detected in the tested cloacal swabs taken from the leatherback sea turtles [33]. Another investigation conducted several years later in this country compared the prevalence of *Salmonella* in leatherback sea turtles with that of green and hawksbill sea turtles [30]. The results indicated a higher prevalence of *Salmonella* in nesting leatherback sea turtles compared to hawksbill sea turtles, while no *Salmonella* was detected in green sea turtles. *S. enterica* serovar Montevideo and *S. enterica* serovar Newport were the only ones detected in this study [30]. The reason for the higher prevalence of *Salmonella* in leatherback sea turtles in this study compared to the previous investigation conducted by Dutton et al. (2013) [33] might be due to sampling from different geographical locations in Saint Kitts, human interaction with animals and environment, the age of the animals, and other possible unknown reasons [30] (Table 3).

In a study in Columbia, *S. enterica* serovar Enteritidis and *S. enterica* serovar Typhimurium were identified in the faeces of semi-aquatic turtles; the results of this study demonstrated the presence of turtle-associated *Salmonella* in Colombia, which is an important risk for humans who are exposed to turtles [34] (Table 3).

### 4.3. Europe

In Europe, direct or indirect contact with reptiles has been linked to *Salmonella* infection in humans, although the source of infection is unknown in many cases [48]. Since 2010, studies and reports have been conducted in various European countries related to the prevalence of *Salmonella* in turtles in UK [44], Italy [45], and Spain [8,46] (Table 3) as well as turtle-associated human salmonellosis in Spain [1,17], France [36,37], and Romania [38] (Table 2). In Spain in September 2010 and October 2011, *S. enterica* serovar Paratyphi B var Java and its possible monophasic variant 4,5,12:b:- dT+ were identified in eight and three human cases, respectively. In six of these cases exposure to pet turtles was reported [17]. Although several *Salmonella* serovars can be carried and transmitted by turtles, *S. enterica* serovar Java has been particularly attributed to these reptiles [7]. In this study, exposure to turtles was not reported in all the cases, demonstrating the significant role of indirect transmission of *Salmonella* due to the long-time survival of this pathogen in the environment [17].

Another investigation in Spain showed two outbreaks of human salmonellosis associated with the same turtle type in Barcelona and Castellón; despite a 300-kilometre distance between these two cities, a strong relationship between cases was confirmed by molecular epidemiology techniques [1]. The authors proposed that a considerable number of turtle-related salmonellosis cases that occur in humans can be neither investigated nor counted [1]. *Salmonella* contamination was also identified in free-living native (*Emys orbicularis*) turtles as well as the most common pet turtle (*Trachemys scripta elegans*) in another study conducted in Eastern Spain [8]. *S. enterica* serovars Thompson and *S. enterica* serovars Typhimurium were the two predominant serovars in this investigation. The presence of *Salmonella* in the intestinal content of turtles was higher than in the cloacal samples [8]. Previous studies in different parts of the world indicated the prevalence of *Salmonella* contamination in pet turtles ranged from 0 to 72.2% [34,49,50,51] and from 0 to 15.4% in free-living turtles [50,52,53,54,55,56]. It can be hypothesised that the lower level of *Salmonella* shedding in free-living turtles compared to captive and pet turtles might be due to fewer encounters with stress factors [8,57].

During the period between September and October 2013, another study was done in Eastern Spain to identify the rate of *Salmonella* contamination of turtles in pet stores and in turtles belonging to private owners [46]. The rate of *Salmonella* isolation was higher in pet store turtles (75%) than in turtles that belonged to private owners (29%). *S. enterica* serovars Typhimurium and Pomona were the most frequently detected serovars among 18 different identified serovars. This big difference in the prevalence of *Salmonella* could be partly explained by the fact that shedding of *Salmonella* might be lower in turtles owned by private owners because of lower exposure to stress factors [47]. The shedding of *Salmonella* from the gastrointestinal tract to the environment is facilitated by stress caused by transport, overcrowding at pet stores, or incorrect and inadequate hygiene [58].

In France, no information has been reported on exposures related to salmonellosis risk through the surveillance system. This information is usually elicited from an investigation into the occurrence of a cluster. Hence, the occurrence of reptile-associated human salmonellosis in France may be underestimated [48]. Recently, a report indicated that two cases of *Salmonella*–rotavirus co-infection have been attributed to the presence of pet turtles [36]. Pet turtles were also identified as responsible for a case of meningitis caused by *S. enterica* subsp. *enterica* serovar Vitkin in a 1-month-old child. Although this *Salmonella* serovar is a common inhabitant in the intestinal tract of reptiles, it has rarely been reported in human cases. Young infants or immunocompromised individuals who have intimate associations with reptiles might be infected with this *Salmonella* serovar [37].

In 2017 in Romania, a case of otitis caused by *Salmonella enterica* subsp. *arizonae* was reported in a 16-year-old immunocompromised boy after he bathed in a lake. This *Salmonella* subsp. is rarely a cause of human infection, although it is a common gut inhabitant of reptiles, such as snakes and turtles. Whilst gastroenteritis is the common clinical symptom of this *Salmonella*, other manifestations, including otitis, mastoiditis, meningitis, osteomyelitis, osteoarthritis, or septicemia, can occur, particularly in young children and immunocompromised individuals [38].

### 4.4. Asia and Oceania

A few studies regarding the *Salmonella* contamination of turtles were undertaken in South Korea [40,41,42]. In an investigation conducted by Back et al. (2016), half of the tested turtles were contaminated with *Salmonella* [42]. Another recent study also confirmed *Salmonella* contamination of popular pet turtle species randomly purchased from pet shops and online markets in Seoul. The recovered *Salmonella* isolates were attributed to nine different serovars [40]. The results of these studies indicated that pet turtles could be a potential risk for human salmonellosis in Korea [42]. In China, *Salmonella* contamination was additionally shown in soft shelled terrapins and pet turtles, which emphasised their role in the risk of human salmonellosis through handling and consumption of turtles [43] (Table 3). Two cases of human salmonellosis caused by *S. enterica* serovar Poona and *S. enterica* serovar Abony have been also reported in Japan due to the exposure of two children to pet turtles [39] (Table 2).

## 5. Conclusions

*Salmonella* is one of the major zoonotic pathogen, which is recognized as a natural inhabitant of the turtle gastrointestinal tract [23]. Turtles can be infected with *Salmonella* throughout their lives, even if they are *Salmonella*-free at the time of sale [7]. There are several routes through which turtles can be infected with *Salmonella*, such as cross-contamination during shipping or through contaminated food and water [23]. Moreover, environment also plays significant role in *Salmonella* contamination of turtles; for instance the soil itself can contain *Salmonella.* This pathogen also has the ability to survive and penetrate through the turtle eggs [7,59]. Stressful conditions might increase the level of *Salmonella* shedding in the turtle’s environment [23]. Therefore, the risk of turtles as a source of human salmonellosis should not be underestimated, particularly the hazard of small pet turtles for young children. Hygiene practices of younger children may also contribute to increased transmission risks in households [12]. Although the authorized sale of pet turtles is prohibited in some countries, such as the United States, turtle-associated *Salmonella* is still a public health concern in many countries. Therefore, reducing turtles’ stress to minimise *Salmonella* shedding [23] as well as providing client education handouts at the points of sale of these animals on correct animal husbandry procedures and hygiene techniques, might reduce the risk of transmission of this significant pathogen to humans [60].

## Figures and Tables

**Table 1:** Most popular pet turtles according to the Reptiles Magazine.

Scientific Name	Common Name	Adult Size (Inches)	Origin
*Trachemys scripta elegans*	Red-Eared Slider	8 to 10	The United States, Asia and Europe
*Terrapene carolina carolina*	Eastern Box Turtle	4 to 8	The United States, Mexico
*Chrysemys picta bellii*	Western Painted Turtle	7 to 8	The United States and Canada
*Graptemys geographica*	Map Turtle	6 to 10	The United States and Canada
*Glyptemys (Clemmys) insculpta*	Wood Turtle	5 to 9	The United States and Canada

**Table 2:** Turtle-associated human salmonellosis in different countries in the last ten years.

Country	Year	Outbreak/ Case Report	Age Range or Median Patient’s Age	No. of Infected Cases	No. of Hospitalization	No. of Death	Source	*Salmonella* Serovar(s)	Reference
**USA**	2017	A multistate outbreak	21 years	76	30	-	Turtles were from street or roadside vendor, a retail store, and festivals.	*S. enterica* subsp. *enterica* serovar Abgeni	[35]
**USA**	2015	Four multistate outbreaks	Children aged <5 years	143	39	-	Small turtles purchased from flea markets or street vendors.	*S. enterica* subsp. *enterica* serovars Sandiego, Poona, Pomona	[10]
**USA**	2011–14	Ten multistate outbreaks	6 years	645	99	-		*S. enterica* subsp. *enterica* serovars Paratyphi B var. Java, Sandiego, Newport, Pomona, Poona, Typhimurium, I 4,[5],12:i-	[23]
**USA**	2014	A multistate outbreak	5 years	40	8	-	Small turtles (<4 inches).	*S. enterica* subsp. *enterica* serovar Poona	[20]
**Chile**	-	Case report in three infants	-	3	-	-	Pet turtles	*S. enterica* subsp. *enterica* serovars Montevideo, Newport, Pomona	[29]
**Spain**	2009	Outbreak	11 months and 4 years	2	-	-	Freshwater turtles (Trachemys scripta troosti) purchased from the same pet-shop.	*S. enterica* subsp. *enterica* serovars paratyphi B var. Java.	[1]
			11 month	1	-	-			
**Spain**	2010–11	Outbreak	Mostly three months to ten years	11	-	-	Turtles	*S. enterica* subsp. *enterica* serovars Paratyphi B var Java, Paratyphi B var Java monophasic variant 4,5,12:b:- dT+. and Paratyphi B sensu stricto	[17]
**France**	-	Case report	-	2	-	-	Turtles kept at home	*Salmonella*–rotavirus co-infection	[36]
**France**	-	Case report	1-month-old infant	1	-	-	Pet turtle	*S. enterica* subsp. *enterica* serovar Vitkin	[37]
**Romania**	-	Case report	16-year-old boy	1	-	-	Turtles in the lake	*S. enterica* subsp. *arizonae*	[38]
**Japan**	2007–8	Case report	5-year-old boy	1	-	-	Turtle kept at the patient’s home	*S. enterica* subsp. *enterica* serovar Poona	[39]
			4-year-old boy	1	-	-	Tortoise kept at the patient’s home	*S. enterica* subsp. *enterica* serovar Abony	

**Table 3:** *Salmonella* recovery from different turtle species around the world in the last ten years.

Country	Turtle Species	Turtle’s Source	Sample	Sample Size	No. of Positive (%)	*Salmonella* Serovar(s)	Reference
**Korea**	Six commercially popular species: Chinese stripe-necked turtles (*Ocadia sinensis*), River cooters (*Pseudemys concinna concinna*), Yellow-bellied sliders (*Trachemys scripta scripta*), Common musk turtles (*Sternotherus odoratus*),Western painted turtles (*Chrysemys picta belli*), Northern Chinese softshell turtles (*Pelodiscus maackii*)	Nine pet shops and eight online markets	Fecal samples	59	35 (59.3)	*S. enterica* subsp. *enterica* serovars Pomona, Paratyphi, Typhimurium, Thompson, Stanley, Braenderup, Kentucky, Singapore, and Potsdam	[40]
**Korea**	Six commercially popular species:Chinese stripe-necked turtles (*Ocadia sinensis*), yellow-bellied sliders (T*rachemys scripta scripta*), River cooters (*Pseudemys concinna concinna*), Northern Chinese softshell turtles (*Pelodiscus maackii*), Western painted turtles (*Chrysemys picta belli*) and common musk turtles (*Sternotherus odoratus*)	Different pet shops and online markets	Fecal samples	35	21 (60.0%)	*S. enterica* subsp. *enterica*	[41]
**Korea**	Six commercially popular species: Chinese stripe-necked turtles (*Ocadia sinensis*), yellow belly sliders (*Trachemysscripta scripta*), river cooters (*Pseudemys concinna* *concinna*), northern Chinese softshell turtles (*Pelodiscusmaackii*), western painted turtles (*Chrysemys picta* *belli*) and common musk turtles (*Sternotherus odoratus*)	Nine pet shops and eight online markets	Fecal samples	34	17 (50.0%)	*S. enterica* subsp. *enterica*	[42]
**China**	Soft-shelled terrapins	Supermarkets and farmer’s markets	Fecal samples	172	51 (29.7%)	*S. enterica* subsp. *enterica.* belonged to twenty-two serovars including Thompson, Hvittingfoss, Typhimurium, Wandsworth, Virchow, Stanley, Saintpaul, Singapore, Kedougou and other subtypes	[43]
	pet turtles			164	31 (18.9%)		
**UK**	Tortoises	Veterinary practice	Cloacal swabs	89	5 (5.6)	*S. enterica* Group D	[44]
**Italy**	*Testudinidae, Trachemys scripta*	Reptile owners	Cloacal swabs	10	3 (30)	*Salmonella* spp.	[45]
**Spain**	Thirty five turtle species	Pet stores and	Water samples	120	24 (20)	Eighteen different serovars belonged to *S. enterica* subsp. *enterica* including Typhimurium and Pomona	[46]
		Private owners		120	96 (80)		
**Spain**	Free-living native (*Emys orbicularis*) and exotic (*Trachemys scripta elegans*) turtles	Captured turtles	Water samples from exotic and native turtle containers	200	8.0 ± 2.5	Eight different serovars belonged to *S. enterica* subsp*. enterica* serovars Typhimurium and Thompson *S. enterica* subsp. *salamaeS. enterica* subsp. *diarizonaeS. enterica* subsp. *houtenae*	[8]
			Cloacal swabs from exotic and native turtles	200	3.0 ± 1.5		
			Intestinal content samples from only exotic turtles	117	12.0 ± 3.0		
**Saint Kitts**	Leatherback sea turtles	Sea	Cloacal swabs	9	3 (33.3)	*S. enterica* subsp. *enterica* serovars Montevideo and Newport.	[30]
	Hawksbill sea turtles			14	1 (7.1)		
	Green sea turtles			9	0 (0)		
**Saint Kitts, West Indies**	Leatherback sea turtles (*Dermochelys coriacea*)	Sea	Cloacal swabs	21	3 (14.2)	*S. enterica* subsp. *enterica*	[33]
**Colombia**	Semi-aquatic turtles	-	Fecal samples	110	30 (27%)	*S. enterica* subsp. *enterica* serovars Enteritidis and Typhimurium	[34]
**Australia**	Common long-neck tortoise (*Chelodina longicollis*)	Captive/Wild	Cloacal swabs	19	2 (10.5)	*S. enterica* subsp. *enterica* serovar Typhimurium	[47]
	Murray River turtle (Emydura macquarii)			12	0 (0)		
	Mary River turtle (Elusor macrurus)			2	0 (0)		
	Sawshell turtle (Elseya latisternum)			1	0 (0)		
	Broadshell turtle (Macrochelodina expansa)			1	0 (0)		
	Krefft’s turtle (Emydura krefftii)			1	0 (0)		
	Irwin’s turtle (Elseya irwini)			2	0 (0)		
	Painted turtle (Emydura subglobosa)			2	0 (0)		
